# Changes in soil properties, X-ray-mineral diffractions and infrared-functional groups in bulk soil and fractions following afforestation of farmland, Northeast China

**DOI:** 10.1038/s41598-017-12809-2

**Published:** 2017-10-09

**Authors:** Qiong Wang, Wenjie Wang, Xingyuan He, Qingfu Zheng, Huimei Wang, Yan Wu, Zhaoliang Zhong

**Affiliations:** 1 Northeast Forestry University, Harbin, 150040 China; 2 Northeast Institute of Geography and Agroecology, Chinese Academy of Sciences, Changchun, 130102 China; 3 Inner Mongolia University for the Nationalities, Tongliao, 028000 China

## Abstract

Analysis of soil properties, the compositional traits in bulk soil and different fractions and their responses to afforestation practices may possibly facilitate clarification of the mechanisms underlying soil changes. Soil properties, the compositional functional groups and minerals were determined in the bulk soil and fractions from forests and adjacent farmlands. The afforestation of farmland could induce accumulation of soil organic carbon [SOC] (+18%) and nitrogen [N] (+4%) with pH increase (+4%), and declines in electric conductivity (−15%) and bulk density (−3%). Sand and aggregates [SA] and easily oxidized fraction [EO] mainly contributed to the SOC and N accumulation. Moreover, afforestation-induced changes were observed in O-H & N-H stretching (−26%), feldspar (+52%) and huntite crystallinity (−40%). The changes of soil properties were strongly associated with the changes in functional groups, followed by minerals. Of them, asymmetric COO- & C = O stretching & O-H bending, symmetric COO- stretching, huntite and smectite-vermiculite crystallinity were the key factors responsible for the changes of soil properties. Our findings highlight that degraded farmland afforestation could strongly affect soil properties in the bulk soil, and the changes in fractions (mainly SA and EO) as well as their changes in the compositional traits strongly supported these bulk soil changes.

## Introduction

Afforestation of degraded farmland appears to be a sustainable alternative to forest conservation because it has the potential to provide woody perennials, and is considered as an important option for carbon sequestration and degraded soil improvement^1^. Numerous studies have reported that afforestation of degraded farmland could induce changes in many soil properties, including soil organic carbon (SOC), nitrogen (N), phosphorus (P), base cations (K, Mg, and Ca), C:N ratios and other nutrient, porosity, and bulk density^2–4^, showing that many soil biological and chemical processes, fertilizer preservation, and carbon sequestration could be affected by afforestation. In Northeast China, a large land reclamation has been carried out in 1950s for securing food production for whole China. Soil degradation has long been observed since then^5^, and possibly decreases the grain productivity in this region^6^. Shelterbelt-afforestation has been originally designed for protecting farmland from wind damage, and its possible effects on soil properties are seldom reported in Northeast China. A full check on the possible changes in SOC, fertility and physicochemical properties after a long-term afforestation of degraded farmland is worthy for evaluation of the national forest policy in Northeast China (e.g. Three-North Protection Forest Program), and also for a possible anti-measure proposal to the local degraded soil improvement^7^.

Besides many evaluations on various afforestation practices^2–4^, underlying mechanisms following an afforestation of farmland received still need more attention via soil fractionation-related soil aggregates studies. Soil aggregates are vulnerable to land use changes and managements^8^, and the degradation of aggregates caused by cultivation is responsible for the loss of soil organic matter (SOM)^9,10^. Soil aggregates protect SOM and act as an important reservoir of carbon and other mineral nutrients^11^, and soil aggregates fractionation is efficient in interpreting the effects of land use on soil carbon and nutrient dynamics. There are several physical and chemical methods available to separate soil fractions of different distinct stability. The physical fractionation, by size and density, can isolate the SOC associated predominantly with soil minerals^12^, and also the SOC protected within aggregates due to their three-dimensional architecture^8^. Several wet chemical methods have been developed over the years for addressing the degree of chemical interactions between organic and mineral phases of soil^13^. Integrating physical and chemical methods is effective in isolating soil fractions with different stabilization potentials, such as Zimmermann, *et al*.^14^ proposed physicochemical fractionation, possibly facilitating a better understanding soil structure from the perspective of the physical, chemical, and biological processes controlling SOM decomposition^15^ as well as possible model parameterization^16,17^.

Fourier transform infrared spectroscopy (FTIR) is a useful technique for characterizing chemical compositions, and has been widely used in soil science for comprehensive demonstrations of soil components and their interactions^18,19^. FTIR can also reveal soil fertility conditions and can be used to examine different soil functional group identification (e.g., O-H, N-H and C-H)^20^, and these functional groups were lowest in the acid-insoluble fraction (AI), but peaked in the particulate fraction (PT) and soluble fraction (SB), together with significant positive correlations with the SOC and N in them^16^. Soil mineralogy could influence SOM^21^ and soil nutrient regulations^22^, and X-ray diffraction (XRD) technique has been used in identifying mineral compositions and traits^23^. Together with the FTIR and XRD techniques, ectomycorrhizal influences on particle size, surface structure, mineral crystallinity, functional groups of soil colloids from different soil origins could be elucidated^18^; and the compositional traits of glomalin related soil protein (GRSP) and their differences in different land uses could be determined^24–26^. To date, the compositional variations in soil after afforestation of farmland have not been well-defined using the FTIR and XRD techniques.

In the present study, by using a combination of the FTIR and XRD techniques and soil physicochemical fractionation, we want to provide an insight into the changes in soil compositional traits following afforestation from the perspective of functional groups and mineral crystallinity in the bulk soil and different soil fractions. We hypothesized that afforestation can strongly alter SOC, fertility and physiochemical properties in the bulk soil and different soil fractions, while underlying reasons for these changes are related with the compositional traits of FTIR-functional groups and XRD-mineral features. The objectives of this study were to explore (1) how large afforestation-induced changes in soil properties, and which soil fractions mainly contribute to the changes? (2) what kind of changes were in XRD-mineral features (relative crystallinity) and FTIR-functional groups (relative content), and which soil fractions contribute to these changes? (3) What’s the associations between soil compositional traits and variations of soil properties? This study is expected to provide the underlying mechanisms of changes in soil properties after afforestation of farmland, and the related data will provide supports for the evaluation of afforestation on soil rehabilitation.

## Results

### Changes in soil properties

The percentage of each fraction (weight of each fraction to total soil mass, in percent) was similar between the forest and the farmland. Afforestation did not make changes in the fraction percentage (*p* > 0.05). The maximum percentage was that of AI+EO (about 55% of total soil mass), followed by AI (about 52% of total soil mass), and SA (about 45% of total soil mass), while the minimum percentage were in EO, PT and SB (less than 3% total soil mass) (raw data not shown here).

**Figure 1:**
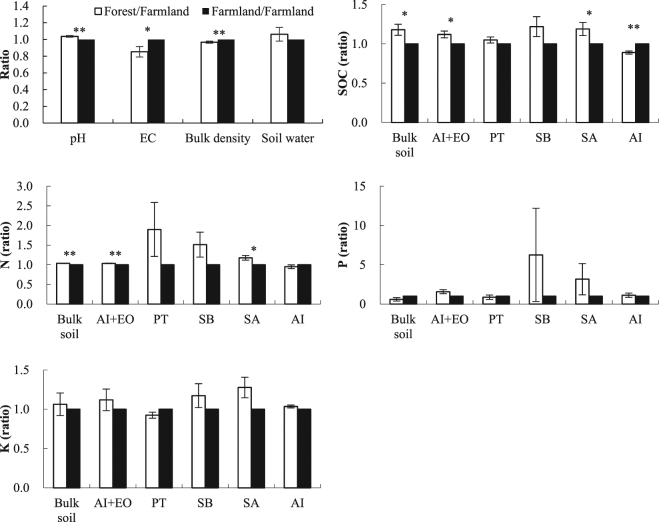
Variations in soil properties in the bulk soil and soil fractions of the forest compared with the farmland (forest/farmland ratio). “**” indicates significant differences between the forest and the farmland in the bulk soil and fractions at *p* < 0.01, while “*” indicates the significant differences at *p* < 0.05.

Variations in soil physicochemical properties between the forest and the farmland are listed in Fig. 1. The afforestation of farmland increased soil pH by 4% (*p* < 0.01) and soil water by 6% (*p* > 0.05), and decreased EC by 15% (*p* < 0.05) and bulk density by 3% (*p* < 0.01).

SOC and fertility changes after the afforestation of farmland are also shown in Fig. 1 and Table S1. On average, in the 6 regions, SOC and N increased by 18% (*p* < 0.05) and 4% (*p* < 0.01), respectively in the forest compared with the farmland, and differences in these increases were found in different soil fractions, with the exception of AI. For example, the AI+EO and SA of the forest showed 4–19% increases in SOC and N (*p* < 0.05), whereas the increasing trends in PT and SB were observed, but not statistically significant (*p* > 0.05). In contrast, there were decreases of 11% for SOC (*p* < 0.01) and 5% for N (*p* > 0.05) in AI associated with afforestation practices. According to the percentage of EO, the afforestation of farmland increased SOC by 116% (*p* < 0.01) and N by 5% (*p* > 0.05) in EO. No significant differences were observed in P and K in the bulk soil or different soil fractions of the forest compared with those of the farmland (*p* > 0.05) (Fig. 1).

### Changes in FTIR-functional groups

Soil functional groups in the forest compared with the farmland are shown in Table 1 and Table S2. Afforestation-induced change was mainly observed in O-H & N-H stretching, with significant decreases of 17–26% in the AI+EO and the bulk soil, and increase of 9% in the PT (*p* < 0.05). Moreover, asymmetric COO- & C = O stretching & O-H bending decreased by 17–21% in the AI+EO (*p* < 0.05). However, afforestation-induced differences in other functional groups were not significant (*p* > 0.05).

**Table 1:** Variations in functional groups in the bulk soil and soil fractions between the forest and the farmland (forest/farmland ratio). “**” indicates significant differences between the forest and the farmland in the bulk soil and soil fractions at *p* < 0.01, while “*” indicates the significant differences at *p* < 0.05. “ns” indicates no significant difference (*p* > 0.05) in the bulk soil and soil fractions of the forest compared with the farmland. Functional groups are Ι: O-H & N-H stretching, ΙΙ: aliphatic C-H stretching, ΙΙΙ: asymmetric COO- & C = O stretching & O-H bending, ΙV: symmetric COO- stretching, V: Si-O-Si & C-O stretching & O-H bending, and VΙ: carbonates.

Functional groups	Bulk soil	Soil fractions				
		AI+EO	AI	SA	PT	SB
Ι	**0**.**74(0**.08)**	**0**.**83(0**.07)*	1.01(0.17)ns	1.15(0.19)ns	**1**.**09(0**.04)*	0.90(0.03)ns
ΙΙ	1.08(0.14)ns	1.01(0.25)ns	0.93(0.15)ns	0.70(0.31)ns	0.93(0.04)ns	1.02(0.09)ns
ΙΙΙ	0.75(0.12)ns	**0**.**79(0**.08)*	1.07(0.22)ns	0.99(0.17)ns	0.90(0.05)ns	1.11(0.12)ns
ΙV	0.82(0.13)ns	0.86(0.12)ns	1.29(0.31)ns	0.92(0.11)ns	1.02(0.09)ns	1.10(0.06)ns
V	0.90(0.07)ns	0.98(0.05)ns	0.95(0.06)ns	1.05(0.09)ns	1.12(0.06)ns	1.08(0.18)ns
VΙ	1.06(0.18)ns	1.11(0.08)ns	0.86(0.12)ns	1.06(0.08)ns	1.15(0.17)ns	0.69(0.11)ns

### Changes in XRD-mineral crystallinity

Soil mineral crystallinity in the forest compared with the farmland are shown in Table 2 and Table S3. Although smectite-vermiculite, cristobalite, quartz, calcite, quartz + illite showed increasing trends in the bulk soil of forest, these differences were not statistically significant (*p* > 0.05). Afforestation-induced significant changes were mainly found in crystallinity of feldspar (+52%) and huntite (−40%) in the bulk soil (*p* < 0.05). In different soil fractions, afforestation markedly increased crystallinity of calcite, smectite-vermiculite and huntite in AI (28%), PT (23%) and AI+EO (22%), respectively (*p* < 0.05). However, afforestation-induced differences in other minerals were not significant (*p* > 0.05).

**Table 2:** Variations in mineral crystallinity in the bulk soil and soil fractions between the forest and the farmland (forest/farmland ratio). “**” indicates significant differences between the forest and the farmland in the bulk soil and soil fractions at *p* < 0.01, while “*” indicates the significant differences at *p* < 0.05. “ns” indicates no significant difference (*p* > 0.05) in the bulk soil and soil fractions of the forest compared with the farmland.

Soil minerals	Bulk soil	Soil Fractions				
		AI+EO	AI	SA	PT	SB
Smectite-Vermiculite	1.35(0.17)ns	0.93(0.09)ns	1.42(0.19)ns	1.19(0.57)ns	**1**.**23(0**.10)*	—
Quartz	1.04(0.12)ns	1.10(0.09)ns	1.03(0.08)ns	1.02(0.19)ns	1.30(0.19)ns	—
Cristobalite	1.30(0.45)ns	1.02(0.17)ns	1.18(0.24)ns	1.06(0.20)ns	1.15(0.16)ns	—
Quartz+Illite	1.02(0.08)ns	1.09(0.05)ns	1.02(0.04)ns	0.92(0.10)ns	1.25(0.13)ns	—
Feldspar	**1**.**52(0**.22)*	0.99(0.23)ns	0.83(0.09)ns	1.27(0.26)ns	0.90(0.15)ns	—
Calcite	1.28(0.28)ns	0.89(0.26)ns	**1**.**28(0**.08)**	0.90(0.28)ns	1.39(0.23)ns	0.90(0.21)ns
Huntite	**0**.**60(0**.10)**	**1**.**22(0**.08)*	—	1.14(0.32)ns	—	2.36(1.28)ns

### Associations between soil properties and soil compositions: Pearson correlation

**Figure 2:**
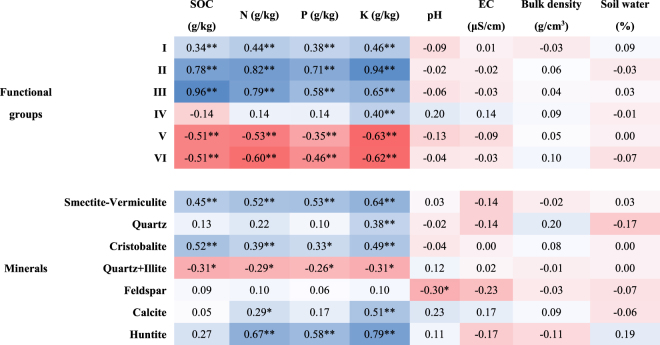
Pearson correlation analysis between soil properties and soil compositions. “**”, “*” respectively mean significance of the correlations at *p* < 0.01 and *p* < 0.05. Functional groups are Ι: O-H & N-H stretching, ΙΙ: aliphatic C-H stretching, ΙΙΙ: asymmetric COO- & C = O stretching & O-H bending, ΙV: symmetric COO- stretching, V: Si-O-Si & C-O stretching & O-H bending, and VΙ: carbonates.

Pearson correlation analysis (Fig. 2) showed that SOC, N, P and K contents were positively correlated with O-H & N-H stretching (r = 0.34–0.46, *p* < 0.01), aliphatic C-H stretching (r = 0.71–0.94, *p* < 0.01), asymmetric COO- & C = O stretching & O-H bending (r = 0.58–0.96, *p* < 0.01) and crystallinity of smectite-vermiculite (r = 0.45–0.64, *p* < 0.01) and cristobalite (r = 0.33–0.52, *p* < 0.05), but negatively correlated with Si-O-Si & C-O stretching & O-H bending (r = −0.35–−0.63, *p* < 0.01), carbonates (r = −0.46–−0.62, *p* < 0.01) and crystallinity of quartz+illite (r = −0.26–−0.31, *p* < 0.05). K content was positively correlated with symmetric COO- stretching and quartz crystallinity. However, in the case of soil physicochemical properties (pH, EC, bulk density, and soil water), only feldspar crystallinity was negatively correlated with soil pH. In all, compared with r values, the folds of higher r values showed that aliphatic C-H stretching and asymmetric COO- & C = O stretching & O-H bending played more roles than the other functional groups and minerals in regulating SOC and fertility.

### Associations between soil properties and soil compositions: Stepwise regression

Stepwise regression analysis confirmed the findings of Pearson correlation analysis, and also highlighted the most probable factors related with soil properties (Table 3). Aliphatic C-H stretching was the first parameter entering into the model for SOC, N, P and K contents, indicating that this functional group was possibly the key factor responsible for SOC and fertility variations. Moreover, stepwise regression and the standard coefficient values showed that SOC, N, P and K were mainly regulated by functional groups, rather than minerals (Table 3). For soil physicochemical properties, symmetric COO- stretching was the only parameter into the model of EC change (*p* = 0.002), showing its possible high contribution to EC change. Bulk density change was possibly driven by both calcite crystallinity and symmetric COO- stretching. Soil pH was mainly regulated by calcite crystallinity. However, there were no obvious associations between soil water and soil compositions related with functional groups and minerals. Consequently, SOC and fertility were mainly affected by various functional groups, and soil physicochemical properties were mainly associated with calcite crystallinity and symmetric COO- stretching.

**Table 3:** Stepwise regression analysis between soil properties (Y) and functional groups and minerals (X) for entering and removing factors. Functional groups are Ι: O-H & N-H stretching, ΙΙ: aliphatic C-H stretching, ΙΙΙ: asymmetric COO- & C = O stretching & O-H bending, ΙV: symmetric COO- stretching, V: Si-O-Si & C-O stretching & O-H bending, and VΙ: carbonates.

Y-factors	X factors	Unstandardized coefficient		Standardized coefficient	T-value	*p*
		B	SE	Beta		
**F-to-enter** ***p*** ≤ **0**.**05**, **F-to remove** ***p*** ≥ **0**.**10**						
SOC (g/kg)	(Constant)	33.818	12.322		2.745	0.010
	ΙΙ	0.281	0.052	0.456	5.441	0.000
	ΙΙΙ	0.070	0.010	0.528	6.633	0.000
	V	−0.011	0.003	−0.181	−3.972	0.000
N (g/kg)	(Constant)	1.778	0.299		5.944	0.000
	ΙΙ	0.025	0.001	0.878	20.153	0.000
	VΙ	−0.004	0.001	−0.245	−5.620	0.000
P (g/kg)	(Constant)	0.051	0.193		0.263	0.794
	ΙΙ	0.012	0.002	0.750	6.413	0.000
K (g/kg)	(Constant)	46.638	3.714		12.559	0.000
	ΙΙ	0.338	0.035	0.862	9.614	0.000
pH	(Constant)	7.526	0.152		49.525	0.000
	Calcite	0.090	0.030	0.470	3.016	0.005
EC (μS/cm)	(Constant)	101.197	15.523		6.519	0.000
	ΙV	0.072	0.021	0.520	3.441	0.002
Bulk density (g/cm^3^)	(Constant)	1.378	0.023		60.416	0.000
	ΙV	0.000	0.000	0.732	3.875	0.001
	Calcite	−0.014	0.006	−0.473	−2.504	0.018

### Associations between soil properties and soil compositions: RDA ordinations

**Figure 3:**
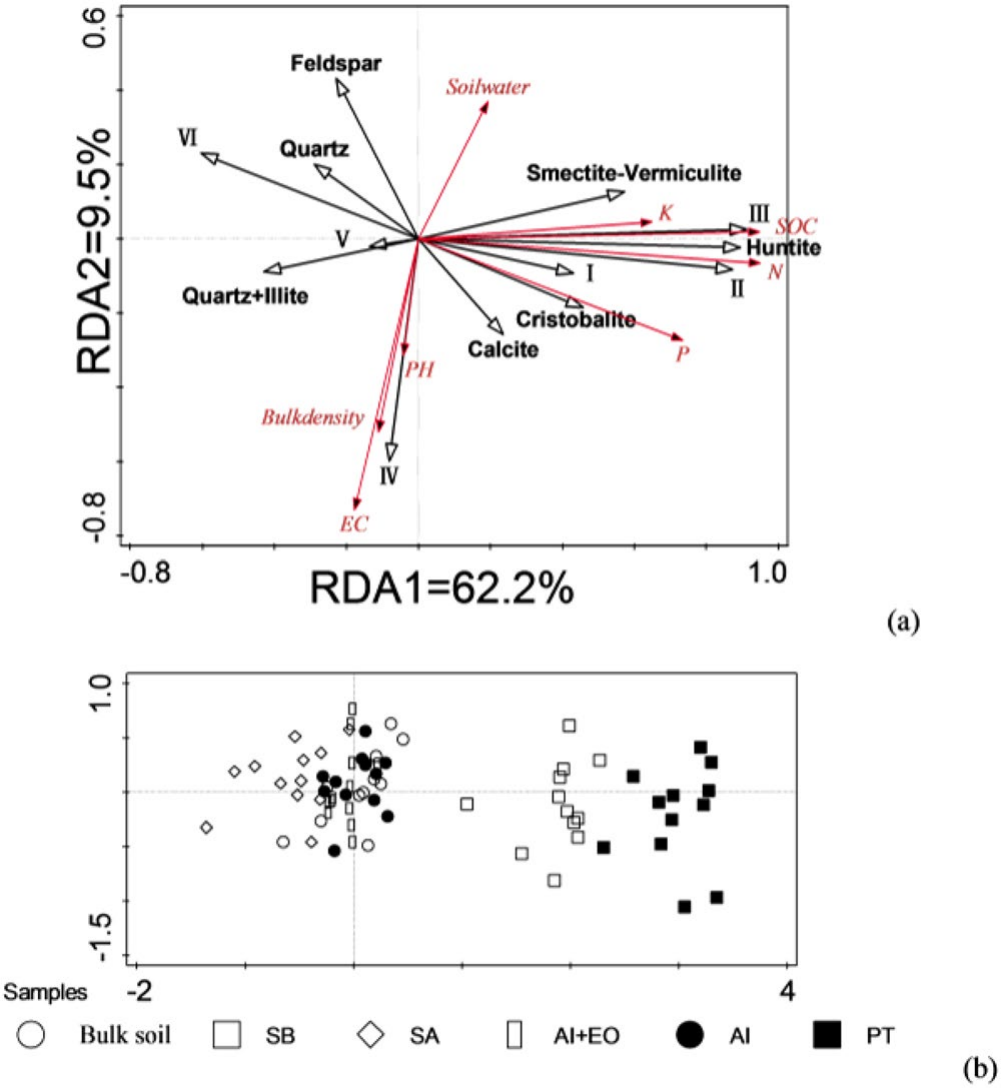
RDA ordination of **(a**) soil properties, functional groups and minerals and (**b**) the bulk soil and different fractions. Soil properties are represented as red lines; soil functional groups and minerals are represented as black lines. Functional groups are Ι: O-H & N-H stretching, ΙΙ: aliphatic C-H stretching, ΙΙΙ: asymmetric COO- & C = O stretching & O-H bending, ΙV: symmetric COO- stretching, V: Si-O-Si & C-O stretching & O-H bending, and VΙ: carbonates.

RDA ordination on soil properties (response factors), functional groups and mineral crystallinity (explanatory factors) (Fig. 3a **)** and explaining percentage from the explanatory factors are listed in Table 4. As shown in Fig. 3a, Two axes explained approximately 71.7% of total variations in soil properties. The higher aliphatic C-H stretching, asymmetric COO- & C = O stretching & O-H bending, huntite crystallinity usually accompanied with the higher SOC, N, P, K contents in soils. The higher amounts in symmetric COO- stretching usually accompanied with higher values in pH, EC and bulk density, and lower soil water content. The contribution of other functional groups and minerals to the variations in soil properties was relatively small (Fig. 3a).

**Table 4:** Explains of functional groups and minerals for soil properties based on RDA. Functional groups are Ι: O-H & N-H stretching, ΙΙ: aliphatic C-H stretching, ΙΙΙ: asymmetric COO- & C = O stretching & O-H bending, ΙV: symmetric COO- stretching, V: Si-O-Si & C-O stretching & O-H bending, and VΙ: carbonates.

Name	Explains (%)	pseudo-F	*p*	*p* (adjusted)
**Simple Term Effects:**				
*ΙΙΙ*	***51***.***7***	***34***.***3***	***0***.***002***	***0***.***009***
*Huntite*	***49***.***8***	***31***.***8***	***0***.***002***	***0***.***009***
*ΙΙ*	***48***.***1***	***29***.***7***	***0***.***002***	***0***.***009***
*VΙ*	***24***.***3***	***10***.***3***	***0***.***004***	***0***.***010***
*Smectite-Vermiculite*	***22***.***7***	***9***.***4***	***0***.***004***	***0***.***010***
*Cristobalite*	***14***.***2***	***5***.***3***	***0***.***022***	***0***.***041***
*Ι*	***12***.***9***	***4***.***8***	***0***.***008***	***0***.***017***
*Quartz*+*Illite*	***11***.***6***	***4***.***2***	***0***.***028***	***0***.***046***
Quartz	6.1	2.1	0.070	0.101
Calcite	5.7	1.9	0.144	0.175
Feldspar	5.3	1.8	0.174	0.189
ΙV	4.9	1.6	0.148	0.175
V	2.4	0.8	0.504	0.504
**Conditional Term Effects:**				
*ΙΙΙ*	***51***.***7***	***34***.***3***	***0***.***002***	***0***.***013***
*Huntite*	***5***.***4***	***3***.***9***	***0***.***008***	***0***.***026***
*ΙV*	***5***.***1***	***4***.***0***	***0***.***002***	***0***.***013***
*Smectite-Vermiculite*	***4***.***0***	***3***.***5***	***0***.***006***	***0***.***026***
Calcite	2.3	2.1	0.098	0.166
ΙΙ	2.1	2.0	0.098	0.166
VΙ	2.4	2.4	0.060	0.156
Ι	1.9	1.9	0.102	0.166
Cristobalite	1.0	1.0	0.422	0.610
Quartz+Illite	0.7	0.7	0.634	0.702
V	0.6	0.6	0.642	0.702
Quartz	0.6	0.5	0.728	0.728
Feldspar	0.7	0.6	0.648	0.702

As shown in Fig. 3b, the RDA ordination of the bulk soil and soil fractions showed PT and SB separated from others. In the direction of RDA1 axis, the higher amounts of these two fractions were in line with the higher aliphatic C-H stretching, asymmetric COO- & C = O stretching & O-H bending, huntite crystallinity, SOC, N, P, and K concentration. In the direction of RDA2, RDA ordination of fractions did not show clearly differences in different soil samples (Fig. 3b).

By using whole variations of all soil properties (fertility, SOC and physicochemical properties) as 100%, the relative contribution of each explanatory factor to the variations in response factors were calculated by the RDA forward selections (Table 4). For simple term effects, we found 8 factors (asymmetric COO- & C = O stretching & O-H bending > huntite crystallinity > aliphatic C-H stretching > carbonates > smectite-vermiculite crystallinity > cristobalite crystallinity > O-H and N-H stretching > quartz+illite crystallinity) gave significant contribution for the variations of response factors (*p* < 0.05), and the explained total variation from them ranged from 11.6% to 51.7%. Conditional effects were also excluded when pooling whole explanatory factors at the same time, the greatest part of the explanation were from only 4 factors at statistical significant level (*p* < 0.05), i.e. asymmetric COO- & C = O stretching & O-H bending (51.7%), huntite crystallinity (5.4%), symmetric COO- stretching (5.1%) and smectite-vermiculite crystallinity (4.0%) (Table 4).

## Discussion

### Changes in soil properties and which soil fractions are susceptible to afforestation?

The afforestation of farmland soils have been reported to induce changes in many soil properties^2,27^. How afforestation changed various soil properties is a hot spot in recent year, and a large field survey together with detailed laboratory measurement could determine the possible soil properties susceptible to afforestation practices in degraded farmland. In this paper, we proved that SOC, N, pH, EC and bulk density, rather than P, K and soil water, were susceptible to afforestation. Afforestation could alter soil properties, and the alteration will facilitate the exact evaluation of afforestation practices and possible soil management.

Firstly, increases of soil pH with sharp declines in EC and bulk density were a feature of the afforestation-induced physiochemical changes in Northeast China. Decrease in soil pH has been reported from earlier afforestation study, with 15% decrease in soil EC^28^. Recently, Rytter^29^ found that soil pH change after five years cultivation of Salicaceae species on former arable soils was about 0.1 units. Our study in this paper found that afforestation increased soil pH by 4%, 0.25 units changes from 7.83 to 8.08. A possible reason for this pattern is that most of Na^+^ in the inland saline-alkali land in Northeast China is stored in deep soil layers, and trees with deep roots could relocate some of the Na^+^ from deep soils to surface soils. The Na^+^ accumulation in surface soils possible resulted in pH increases^30^. Moreover, the tree canopy coverage could decrease the direct evapotranspiration from soil surface compared with farmland crops, and thus the vertical movement of various salt from deep soil to surface soils was declined. Especially, the soil physical improvement (bulk density decreased by 3%) could also make it possible for salt infiltration from surface to deep soils^30^. In the case of farmland, most biomass was manually removed or fired, while forest-induced larger litter returning to both surface and deep soils may result in looser soil existence. Our finding is consistent with other studies, e.g. 5.7 mg/cm^3^ year decrease in bulk density in the surface soil layer (0–20 cm) from returning farmland to larch (*Larix gmelini*) plantations in Northeast China^7^, bulk density decrease under 12 year old plantations with Salicaceae^31^.

Secondly, afforestation markedly induced 18% SOC (3.81 g/kg) and 4% N (0.06 g/kg) accrual, while no changes were found in P and K, which is another feature of the afforestation-induced soil nutrient changes in Northeast China. Different impacts of afforestation on SOC and N dynamics have been reported^32,33^. The carbon sequestration expected to result from the large-scale afforestation projects is a worthy goal, but occasionally these projects have negative impacts on ecosystem health^27^. Cong, *et al*.^34^ reported that compared to arable land, 5 years of afforestation caused a decrease in SOC and N concentration, whereas 25 years of afforestation resulted in an increase in SOC content. SOC increased by 5.2 g/kg and 10.4 g/kg, whereas total N significantly increased 0.2 g/kg to 0.6 g/kg after nearly 40 year afforested Hippophae (*Hippophae rhamnoides*) and afforested Robinia (*Robinia pseudoacacia*) of former arable lands, respectively^35^. Consequently, the year of afforestation and tree species were two key factors for SOC and N changes. In this study, poplar afforestation was initiated in 1970s. The nearly 40 years of afforestation could result in SOC and N accrual, and this result supports the ecological evaluation of the large afforestation program in China, such as the Three-North Forest Protection Program^36^.

Thirdly, the most probable soil fractions responsible for the SOC and N alternation in the bulk soil were confirmed in this study, and showing risk management for SOC and N storage. Owing to the fact that different soil fractions have different SOC and N protecting mechanism, this identification will favor the mechanical understanding of afforestation-induced SOC and N alternation. Soil fractions in this paper can be grouped into two labile and active fractions (PT and SB), one intermediate fraction (SA), and two resistant and passive fractions (AI+EO and AI)^37^. The first two fractions generally had more rapid turnover than bulk soil^14^, and in the present study, afforestation increased SOC by 5–22%, N by 51–90% in PT and SB. However, these increases were not statistically significant (*p* > 0.05) owing to large site-variations were observed in this study. In the case of SA (percentage was about 45%), SOC and N respectively increased by 19% and 18% following afforestation, showing greater protection of SOC and N in SA in forest soils. Since SA also comprises PT attached to sand grains (heavy fraction), it also can be characterized as active pool with a short turnover time^38^. Our finding confirmed that SA could respond to afforestation due to higher sensitivity^39^. Soil AI was a chemically resistant fraction, which was extracted from the silt and clay fraction^14^ with approximately 52% of total bulk soil. In this paper, we found that afforestation could lose SOC and N in AI compared with the adjacent farmland, possible owing to the fact of more original farmland-derived SOC loss, but less forest-derived SOC input in the AI after afforestation. Interestingly, SOC and N increased by 4–12% in AI+EO, showing EO adhere to silt and clay has greatly increased, which may finally resulted in the accrual of SOC and N in silt and clay of the bulk soil following afforestation. EO is a more sensitive indicator of changes in SOC resulting from different management practices than total organic carbon^40^, and has higher SOC concentrations than some stable fractions^41^. Wang, *et al*.^42^ suggested that conversion of cultivated forest to cropland decreased the total organic carbon content, the converting forest land to cropland may cause long-term carbon sequestration because the mineral-associated organic carbon is the major portion of stable organic carbon in soils. Our findings confirmed that afforestation can protect SOC and N in SA, result in an activation of resistant fraction (AI) into the recent C and N cycle, and SOC and N accrual might face the risk of SOC and N stability changes following afforestation.

### Changes in FTIR- and XRD-related soil compositional traits and the most affected fractions to afforestation

Change of FTIR-related functional group was found the most probably in O-H & N-H stretching in the bulk soil following afforestation, i.e., relative amount of O-H & N-H stretching in the forest was 74% of that in the farmland bulk soil. O-H & N-H stretching mainly included stretching of the structural OH of clay minerals and oxides, O-H stretching of sorbed water, O-H stretching of carboxylic acids, phenols, and alcohols, N-H stretching of amines and amides^20^. This kind of O-H & N-H stretching change was in line with a decrease in AI+EO (17%) in the bulk soil. Owing to the fact of non-changes in O-H & N-H stretching in AI (1%), EO should be the key fraction for the decrease and EO is a labile fraction abundant of amino acids, simple carbohydrates, a fraction of microbial biomass, and other simple organic compounds^43^. Changes in functional groups were reported in different land uses^44^, forest types^17^ and tree species^26^ and soil fungi differences^18^, and underlying reason for the changes in functional group may be related to them. Ectomycorrhizal fungi could induce 12–35% decreases in most functional groups in soil colloids^18^ or change soil via GRSP (a glycoprotein abundantly produced on hyphae and spores of arbuscular mycorrhizal fungi)^25^. Main crop for the farmland (maize) and main tree species in the forest (poplar spp.) also different in their returning soil materials, and this kind of differences should be another basis found in functional groups. Furtherly, extracellular enzymes are important in the interaction between soil organic materials and soil fungi, and one study has pointed out that soil fungi with higher enzymatic activities induced larger reduction of functional groups compared with the fungi with lower enzymatic activities^19^. These enzymatic differences both in types and activity may play key roles in the decomposition processes of macromolecular substances in SOM and the infrared functional traits variation^19^. The functional group related SOM resist decomposition because of their chemical composition: they are poor in reactive functional groups that can be readily cleaved by enzymes^45^.

Changes in XRD-minerals crystallinity were mainly observed in feldspar calcite and huntite. Although crystallinity of smectite-vermiculite, cristobalite, quartz, calcite and quartz+illite increased by 2–35%, however, the increases were not statistically significant (*p* > 0.05) owing to large site-variations. Soil mineralogy is an inherent property for soils, dictated by the integrated effects of the soil forming factors and soil environmental conditions, and little affected by short-term managements and land use changes^46^. However, several lines of inquiry challenge this dogma, e.g. soil minerals are susceptible to enhanced weathering under acid conditions^47^. Feldspar is a group of minerals that are very important in rock formation, accounting for over half the earth’s crust and including SiO_2_, Al_2_O_3_, K_2_O, Fe_2_O_3_, Na_2_O, CaO^48^, while weathering of feldspar led to the information of minerals such as kaolinite^49^, illite^50^ or mixed-layer clay minerals^51^. Afforestation significantly increased feldspar crystallinity by 52%, and soil SA (+27%) possibly contributed to this increase. Gleeson, *et al*.^52^ found that bacterial communities preferentially inhabit minerals with specific inorganic nutrient contents such as plagioclase and K-feldspar (rich in Mg, Ca, K). Explanation of the increased feldspar after the afforestation of farmland may due to feldspar wrapped in aggregates (e.g. SA) and bacterial communities, and further resisted feldspar decomposition and weathering. In addition, afforestation markedly decreased huntite (Mg_3_Ca(CO_3_)_4_) crystallinity by 40%, which is categorized in the group of salt-type carbonate minerals. It caves mineral undergoes diagenetic processes as dissolution, recrystallization, micritization or even dolomitization^53^. Especially, huntite is insoluble in water (sediment), and forest root-organic secretions into deep soils are possibly decompose the mineral, and improve plant availability together with help from soil fungi. Furthermore, calcite (CaCO_3_, usually expressed as inorganic carbon) has been proved to a large carbon sink of terrestrial ecosystem, and agricultural practices could induce reduction of its storage^54–56^. In the site with abundance of Ca^2+^ ^57^ and CO_2_, refixation of inorganic carbon by formation of CaCO_3_ is possible^57^. With the 40% decline of huntite in the bulk soil and non-existence of humite in AI, we found 1.3-fold increase in calcite, possible owing to this kind of re-fixation of Ca and CO_2_.

### Afforestation-induced changes in soil properties were ascribed to specific functional groups and mineral crystallinity

Shifts of soil compositions may lead to SOC and fertility changes following land use change^17^. In general, SOC and fertility were positively correlated with O-H & N-H stretching, aliphatic C-H stretching and asymmetric COO- & C = O stretching & O-H bending, while negatively correlated with Si-O-Si & C-O stretching & O-H bending and carbonates. Among 6 functional groups, aliphatic C-H stretching was the key factor responsible for the SOC and fertility variations, and both the Pearson correlation and stepwise regression analysis confirmed this. The amount of aliphatic C-H stretching indicates the water affinity of soil organic materials^58^, and the more aliphatic compounds indicate more stability against microbial degradation, rate of wetting, and adsorption processes^59^ as well as more stabilized aliphatic hydrocarbons to SOM^60^. RDA further indicated that asymmetric COO- & C = O stretching & O-H bending explained changes of holistic soil properties by 51.7%, and this functional group mainly existed in salts of carboxylic acids, amides, ketones, sorbed water^20^. Similar to our study, Wang, *et al*.^17^ also suggested the close correlations possibly contributed to the forest-dependent variations in soil C and N dynamics. An explanation is that glomalin-related organic materials and fungi–soil colloids could affect SOC sequestration through the alteration of functional groups^18,61^. The dynamics of microbes and their metabolisms are generally controlled by soil physicochemical properties^62^, while the changes in soil physicochemical properties are in turn associated closely with soil microbial alternations, enzymatic changes or functional group changes^18,19^. For example, soil EC was affected by symmetric COO- stretching of the salts of carboxylic acids (Table 3). Soil salinization and alkalinization is the process of accumulation of free salts in subsoil and groundwater, which leads to soil degradation and hinders the growth of plants^63,64^.

Although soil minerals played relative weaker roles than functional groups in regulating SOC and soil fertility (Fig. 2 and Table 3), their importance should not be ignored^17,18^. Soil minerals play vital roles in soil fertility and SOC accumulation since mineral surface serve as potential sites for nutrient storage and soil minerals serve as core component for aggregates formation^22^. Except feldspar and quartz, close correlations were found between smectite-vermiculite, cristobalite, calcite, huntite, quartz+illite and SOC and soil fertility (Fig. 2). Smectite can form organoclay, enhancing the retention of organic matter in soil^65^. Vermiculite is a hydrated magnesium-aluminum-iron silicate^66^, which can absorb such liquids as fertilizers, herbicides, and insecticides^67^. Illite/smectite clay minerals contained in the soils have a great capacity to retain and supply large quantities of nutrients, such as Ca, Mg, K, and NH_4_ which tend to favor high soil fertility^68^. Calcite is the most stable polymorph of CaCO_3_ under ambient condition, and is ubiquitously found in various surface environments including under reducing conditions^69^. Quartz and cristobalite have the same chemical formula (SiO_2_), but they have different crystal structure^70^. The crystal structure of cristobalite was responsible for improving SOC and soil fertility. In the case of soil physicochemical properties, calcite (CaCO_3_) was positively correlated with soil pH. CaCO_3_ dissolves in hydrochloric acid, for example, CaCO_3_ + 2HCl = CaCl_2_ + H_2_O + CO_2_, or a reverse process for calcite formation^71,72^. Therefore, the higher soil pH (alkaline environment) with atmosphere Ca deposition and higher respiratory CO_2_ in soil usually accompanied the higher calcite accumulation in soil^72^.

## Conclusions

The afforestation of farmland decreased EC and bulk density, and increased SOC and N, rather than soil water, P, K. For the SOC and N accumulation, SA and EO mainly contributed to the changes, and the declines in AI showed a possible risk for the shorter storage of carbon during the afforestation of farmland. Together with these findings, changes of O-H & N-H stretching and crystallinity of feldspar, huntite and calcite were susceptible to afforestation, and EO mainly contribute to O-H & N-H stretching (the most probable functional group susceptible for afforestation) change. Moreover, asymmetric COO- & C = O stretching & O-H bending, huntite, symmetric COO- stretching and smectite-vermiculite were the 4 key factors responsible for the variations of soil properties. In this study, an insight into the changes in soil compositional traits following afforestation from the perspective of functional group and mineral crystallinity changes in the bulk soil and different soil fractions.

## Materials and Methods

### Natural conditions of the study regions

Songnen Plain is located in the middle of Northeast China and crosses Heilongjiang Province, Jilin Province, and Inner Mongolia Autonomous Region, and the total area is 182,800 km^2^. Basic data for the poplar plantation forests are the following: forest age: 17–24 years; geographic coordinates: 124°19′–126°51′N, 45°08′–46°55′E; altitude: 146–400 m; mean annual temperature: 3.45 °C; mean annual precipitation: 456.67 mm; tree trunk diameter at breast height: 19.80–28.50 cm; and tree height: 12.40–20.60 m.

### Preparation of soil samples

In total, soil samples were collected from 72 poplar plantation forests and the adjacent farmland (principal crops: corn) in 6 regions (Zhaodong, Lanling, Dumeng, Zhaozhou, Fuyu, Mingshui) distributed in Songnen Plain (6 regions × 12 sites × 2 land-use types = 144 samples). The soils are typical black soils, including Chernozem, Phaeozem, Arenosols, and Cambisols, and some degraded Solonetz^73^. In each of the 6 regions, soil samples were collected from the 0–20 cm soil layer using a 100-cm^3^ cutting ring. All samples were collected from June to August, 2012. After fully air-drying and removing small stones, distinguishable plant roots and other debris, samples were passed through a 2-mm sieve and used for subsequent laboratory analyses.

### Soil physicochemical fractionation

**Figure 4:**
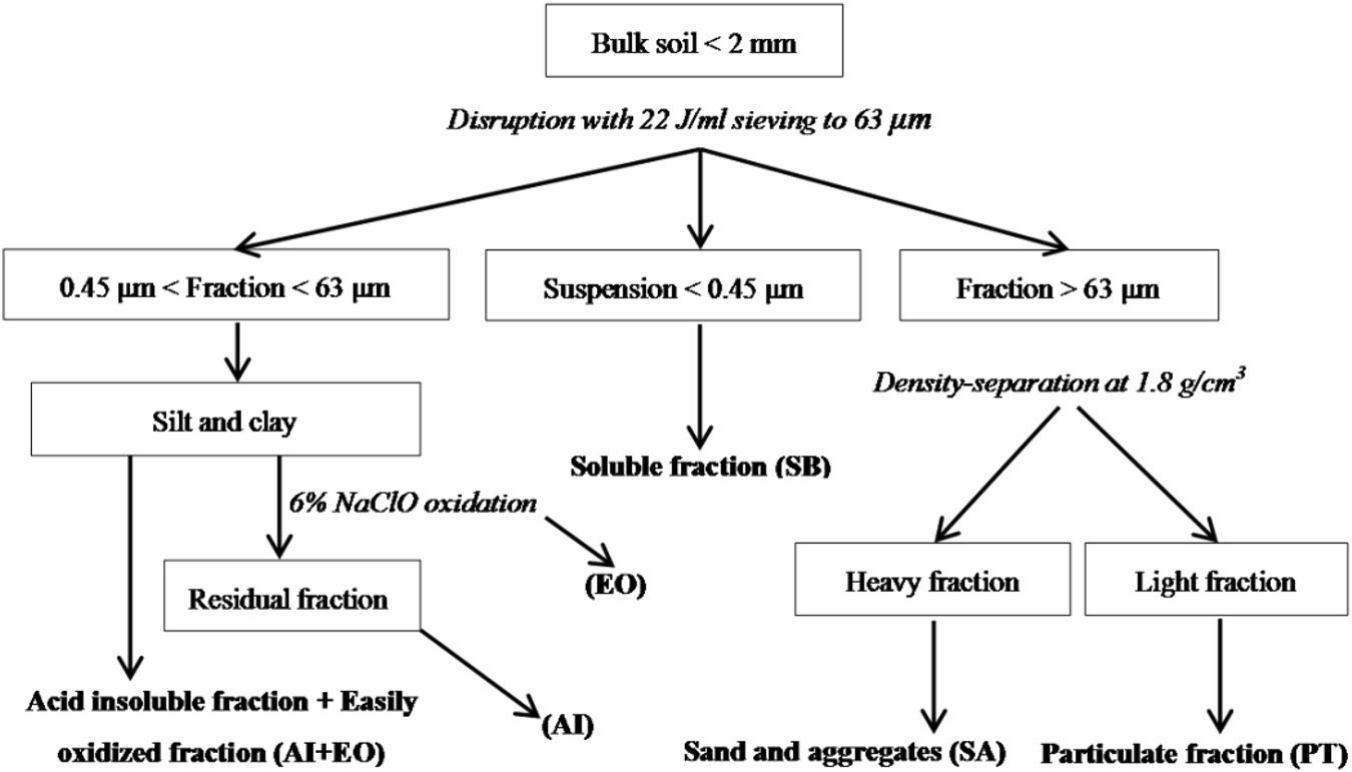
Flow chart of soil fractions based on particle components.

Soil fractions with different physicochemical stabilities were separated using the physicochemical method described by Zimmermann, *et al*.^14^ (Fig. 4). Poplar forests (farmland) soil samples of 12 sites from the same region (Dumeng, Fuyu, Lanling, Mingshui, Zhaodong and Zhaozhou) were mixed to form a composite sample to separate soil fractions (12 composite samples: 6 forests and 6 farmland). Soil samples (< 2 mm particle size) were mixed with water in a 1:5 soil:water ratio and dispersed for 1 min using a probe-type ultrasonic vibrator (SCIENTZ-IID, China). The dispersed suspension was wet sieved through a 63-μm sieve until the rinsing water was clear. The soil fraction with particle size > 63 μm remained on the sieve and that with particle size < 63 μm was filtered. The soil was separated by stirring the > 63-μm fraction with a 1.8 g/cm^3^ NaI solution. The material that remained in suspension was designated the particulate fraction (PT) and the material that settled was designated as the sand and aggregate (SA) fraction. The < 63-μm soil fraction was centrifuged, and the supernatant was collected by suction filtration (using a 0.45-μm filter membrane). The soluble fraction (SB) was freeze-dried from the filtered solution (Scientz-10N; Ningbo Scientz Biotechnology Co., Ltd., China), and solid particles ( > 0.45 μm) comprising silt and clay were oxidized by sodium hypochlorite (6% NaClO). Residual material was designated as the acid-insoluble fraction (AI). The easily oxidized fraction (EO) was calculated as the difference between silt and clay and AI (Fig. 4).

### Determination of soil properties

All 144 soil samples were measured for all physicochemical properties described herein. Soil pH was measured in a solution of 1.00 g soil sample in 5 ml deionized water using a precise pH meter (Sartorius PB10; Sartorius, Germany). Soil electrical conductivity (EC) was determined from the same solution using an EC meter (DDS-307; Shanghai Precision Scientific Instruments Co., Ltd., China). Soil bulk density was calculated as the ratio between the air-dried soil mass and the soil volume (400 cm^3^, which was fixed by the soil cutting ring). Soil water was calculated as (fresh weight – dry weight)/dry weight × 100%. SOC, N, P and K of the bulk soil and 5 soil fractions (AI, AI+EO, SA, PT and SB) in 12 composite samples were measured. SOC content was determined using the potassium dichromate volumetric method (external heating method). N content was determined using the semi-micro Kjeldahl method. P and K contents were determined using the sodium hydroxide melt method. All the related methods were detailedly described by Bao^74^.

### FTIR analysis

**Figure 5:**
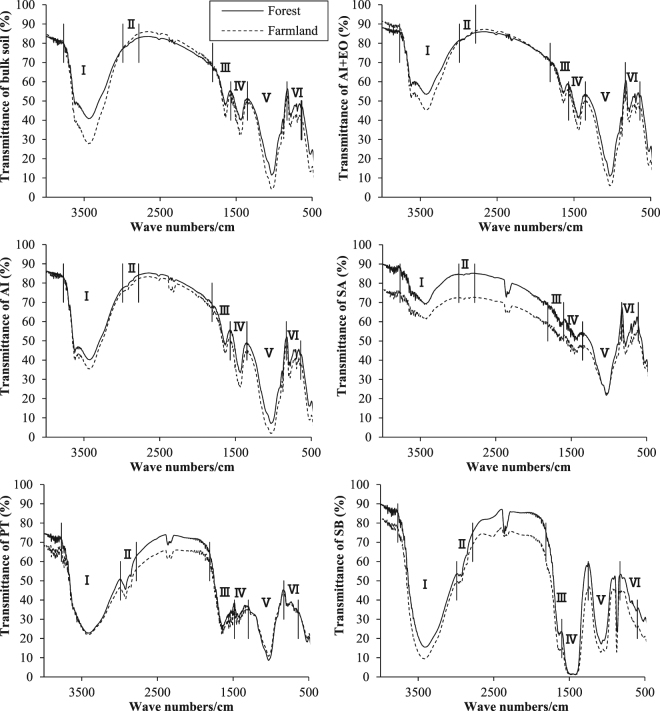
FTIR of functional group in the bulk soil and soil fractions between the forest and the farmland. Functional groups are Ι: O-H & N-H stretching, ΙΙ: aliphatic C-H stretching, ΙΙΙ: asymmetric COO- & C = O stretching and O-H bending, ΙV: symmetric COO- stretching, V: Si-O-Si & C-O stretching and O-H bending, and VΙ: carbonates.

Functional groups of the bulk soil and 5 soil fractions (AI, AI+EO, SA, PT and SB) in 12 composite samples were measured and classified into 6 groups^20^ (Fig. 5). Two milligrams of oven-dried soil sample and 0.20 g oven-dried potassium bromide (KBr) were carefully mixed homogenized agate-milled and ground and then pressed into a sample disk. Afterwards, the disk was immediately put into the sample holder and FTIR spectra (IRAffinity-1; SHIMADZU, Japan) at a wave spectrum of 4,000–500 cm^−1^ were recorded (background correction). Functional group Ι included stretching of the structural OH of clay minerals and oxides, O-H stretching of sorbed water, O-H stretching of carboxylic acids, phenols, and alcohols, N-H stretching of amines and amides (hereafter abbreviated as O-H & N-H stretching). Functional group II included aliphatic C-H stretching (hereafter abbreviated as aliphatic C-H stretching). Functional group III included asymmetric COO- stretching of the salts of carboxylic acids, O-H bending of sorbed water, C = O stretching of carboxylic acids, amides, ketones (hereafter abbreviated as asymmetric COO- & C = O stretching and O-H bending). Functional group IV included symmetric COO- stretching of the salts of carboxylic acids (hereafter abbreviated as symmetric COO- stretching). Functional group V included Si-O-Si stretching of clay minerals and oxides, C-O stretching of polysaccharides, bending of structural OH of clay minerals and oxides, C-O stretching, and O-H bending of -COOH (hereafter abbreviated as Si-O-Si & C-O stretching and O-H bending). Functional group VI included carbonates. All the FTIR images were adjusted to the same size, and Image J software was used to compute the peak area of each functional group. The area reflected the relative content of each functional group^61^.

### XRD analysis

**Figure 6:**
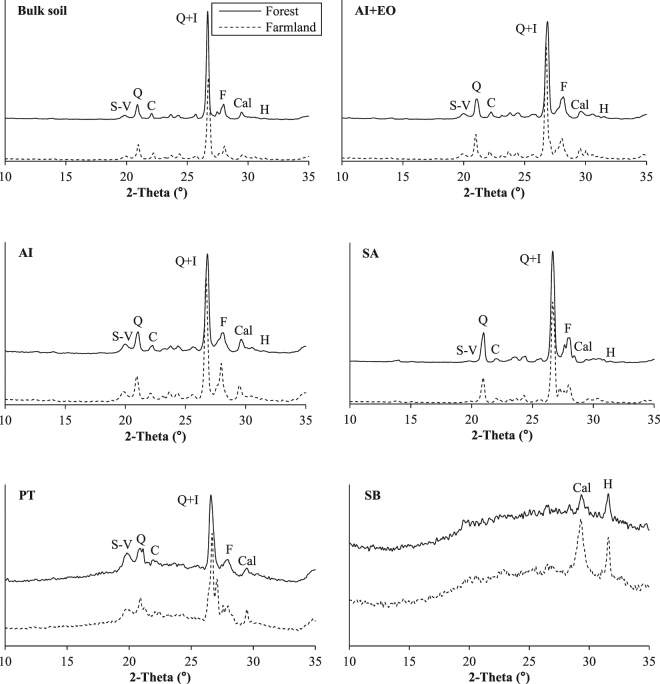
XRD spectrum of minerals in the bulk soil and soil fractions between the forest and the farmland. Minerals are S-V: Smectite-Vermiculite, C: Cristobalite, Q: Quartz, F: Feldspar, Cal: Calcite, H: Huntite, Q+I: Quartz+Illite.

According to the method of Feng, *et al*.^23^, minerals of the bulk soil and 5 soil fractions (AI, AI+EO, SA, PT and SB) in 12 composite samples were measured and divided into 7 major components: smectite-vermiculite, cristobalite, quartz, feldspar, calcite, huntite, quartz+illite (Fig. 6). An XRD meter (D/Max 2200, Rigaku, Japan) equipped with a rotating anode (Philips, Netherlands) was used for XRD analysis. Cu K*α*1 radiation was generated at 30 mA and 40 kV. The range of 2*θ* diffraction angles was 10°–35° with steps of 0.02° and a measuring time of 0.3 s per step. The original data were rectified using the Jade 5.0 software to eliminate K𝛼 and then obtain the XRD pattern for each sample. The upper area (*a*_*c*_), which was separated with the smooth curve connecting each point of minimum intensity, corresponded to the crystalline portion, and the lower area was the background containing the amorphous portion (*a*_*b*_). The Jade 5.0 software was used to calculate relative crystallinity of soil minerals (relative crystallinity =  *a*_*c*_/(*a*_*c*_ + *a*_*b*_) × 100)^18^.

### Data analysis

In order to exclude variations of soil properties, functional groups and minerals relative changes in different regions, values of forest/farmland (representing the relative changes in afforestation relative the adjacent farmland) and farmland/farmland (representing changes of farmland, value = 1) in the bulk soil and soil fractions in 6 regions were effectively applied for analyze the variation in the forest with reference to the farmland. Analysis of variance (ANOVA) with least significance difference (LSD) pairwise comparison was used to identify variations of soil properties, functional groups and minerals in the bulk soil and fractions between the forest and the farmland. Pearson correlation analysis (raw data) was used to examine correlations between soil properties (SOC, N, P, K, pH, EC, bulk density and soil water) and soil compositions (functional groups and minerals) in soils (bulk soil and soil fractions). Step regression analysis (raw data) was used to identify the contribution of different soil compositions to variations in soil properties in the soils. Redundancy analysis (RDA) (raw data) was conducted to examine which soil compositions could markedly contribute to variations in soil properties (Canoco 5.0) in the soils. Data analysis was performed using SPSS 22.0 (IMB, USA) and JMP 11.0 (SAS Institute, Cary, NC).

### Data availability statement

The datasets generated during the current study are available from the corresponding author on reasonable request.

## Electronic supplementary material

Supplementary information
