# Supplementary material for: Changes in soil properties, X-ray-mineral diffractions and infrared-functional groups in bulk soil and fractions following afforestation of farmland, Northeast China
Source: Sci Rep. 2017 Oct 9;7:12829. doi: 10.1038/s41598-017-12809-2 (PMC5634410; doi:10.1038/s41598-017-12809-2)
Supplement: Supplementary file 1 — Supplementary information [file 41598_2017_12809_MOESM1_ESM.pdf]

Changes in soil properties, X-ray-mineral diffraction and infrared-functional groups in bulk soil and fractions following afforestation of farmland, Northeast China

Wang Qiong[2], Wang Wenjie[1, 2*], He Xingyuan[2], Zheng Qingfu[3], Wang Huimei[1], Wu Yan[1], Zhong Zhaoliang[1]

[1]Northeast Forestry University, Harbin 150040, China

[2]Northeast Institute of Geography and Agroecology, Chinese Academy of Sciences, Changchun 130102, China

[3]Inner Mongolia University for the Nationalities, Tongliao 028000, China

[*]Corresponding author

E-mail: wjwang225@hotmail.com

Telephone/Fax: +86-451-82190092, +86-431-85542336

Supplementary information

Table S1. SOC, N, P, K in the bulk soil and soil fractions between the forest and the farmland

Soil properties	Regions	Bulk soil		AI+EO		PT		SB		SA		AI	
		Forest	Farmland	Forest	Farmland	Forest	Farmland	Forest	Farmland	Forest	Farmland	Forest	Farmland
SOC (g/kg)	Lanling	23.14	19.88	12.71	12.27	317.06	317.06	88.00	88.00	8.15	8.06	14.24	16.82
	Zhaodong	20.53	20.53	19.23	18.53	297.65	239.41	101.04	68.44	12.94	8.41	16.18	19.41
	Dumeng	12.06	10.10	21.84	17.27	239.41	232.94	107.56	61.93	5.18	3.88	21.35	22.65
	Zhaozhou	15.97	15.27	21.84	21.49	181.18	180.65	68.44	64.52	8.41	8.00	16.18	17.47
	Fuyu	23.79	19.88	21.84	17.93	297.65	293.53	61.93	58.44	11.65	10.35	20.71	22.00
	Mingshui	39.44	26.40	19.88	17.27	293.53	284.71	94.52	94.52	18.76	17.47	18.12	20.71
N (g/kg)	Lanling	1.58	1.50	1.19	1.18	9.80	5.60	4.20	1.40	0.73	0.59	0.88	1.15
	Zhaodong	1.86	1.78	1.86	1.81	18.20	9.80	9.80	8.40	0.81	0.74	1.33	1.40
	Dumeng	0.81	0.80	1.86	1.83	15.40	22.20	9.80	5.60	0.14	0.11	1.83	1.76
	Zhaozhou	1.61	1.55	1.61	1.53	9.80	16.60	11.40	9.80	0.59	0.42	1.15	1.12
	Fuyu	1.85	1.81	1.90	1.82	21.70	4.20	7.00	7.00	1.05	1.01	1.71	1.60
	Mingshui	2.01	1.95	1.71	1.62	16.80	12.60	9.80	9.80	1.12	1.09	1.23	1.46
P (g/kg)	Lanling	0.06	0.41	0.47	0.25	2.36	3.49	6.99	0.19	0.29	0.70	0.65	0.44
	Zhaodong	0.73	0.88	0.50	0.58	2.98	9.93	0.19	2.87	0.36	0.03	0.46	0.24
	Dumeng	1.10	0.70	0.47	0.70	1.38	9.31	0.04	1.69	0.16	0.16	1.28	0.73
	Zhaozhou	0.59	1.09	0.84	0.46	7.97	3.95	3.28	4.42	0.60	0.21	0.15	0.50
	Fuyu	0.32	0.91	0.63	0.30	2.56	1.95	3.95	6.32	0.20	0.26	0.34	0.46
	Mingshui	0.18	1.02	0.36	0.17	2.15	2.92	0.45	2.98	0.53	0.52	0.32	0.53
K (g/kg)	Lanling	49.46	64.93	66.69	54.00	166.20	206.10	218.80	114.50	50.95	57.97	53.80	52.39
	Zhaodong	62.95	45.59	81.40	47.31	177.40	185.40	190.70	171.30	66.91	48.62	57.51	54.34
	Dumeng	80.33	53.97	52.01	62.70	171.50	197.60	195.20	191.30	69.34	78.03	48.93	44.71

**Zhaozhou**	57.68	74.29	54.98	55.15	190.20	218.00	210.00	186.10	98.54	59.75	44.34	46.38
**Fuyu**	65.36	51.68	57.62	51.14	168.90	172.10	183.10	203.00	97.64	65.10	50.82	49.43
**Mingshui**	47.96	67.94	51.31	63.17	206.50	194.60	189.10	196.50	89.05	65.14	58.79	56.21

Note: particulate fraction [PT], sand and aggregates [SA], soluble fraction [SB], acid insoluble fraction [AI], and silt and clay, i.e. acid-insoluble and easily oxidized fraction [AI+EO], soil organic carbon [SOC], nitrogen [N], phosphorus [P], potassium [K].

Table S2. The relative contents of functional groups in the bulk soil and soil fractions between the forest and the farmland

Functional groups	Regions	Bulk soil		AI+EO		AI		SA		PT		SB	
		Forest	Farmland	Forest	Farmland	Forest	Farmland	Forest	Farmland	Forest	Farmland	Forest	Farmland
O-H & N-H stretching	Lanling	5663	8085	4516	8538	7697	5642	2872	1957	7947	7793	11591	11114
	Zhaodong	6359	7307	3314	4193	2705	8402	1846	2586	8370	8679	9417	10384
	Dumeng	3521	9403	4719	5041	8694	8581	1651	2279	8654	8387	8844	10379
	Zhaozhou	6264	8789	4763	4754	6413	9082	2064	2539	8783	7399	9008	9914
	Fuyu	9478	11144	6481	7162	8980	6946	2835	1555	9652	8253	7244	9214
	Mingshui	8840	9643	5930	7229	9260	6844	3155	2357	8149	7020	9728	10806
Aliphatic C-H stretching	Lanling	22	29	40	51	27	37	33	51	381	399	499	351
	Zhaodong	29	31	12	38	27	59	49	90	375	495	277	261
	Dumeng	22	16	62	44	131	88	88	40	488	509	354	419
	Zhaozhou	35	22	22	33	37	48	16	53	339	346	365	449
	Fuyu	31	40	44	49	66	62	11	59	398	428	400	383
	Mingshui	37	35	44	22	68	64	22	66	451	444	427	443
Asymmetric COO- & C=O stretching & O-H bending	Lanling	317	491	209	479	483	282	106	90	2254	2280	795	583
	Zhaodong	220	387	184	269	181	471	61	130	1690	1802	554	485
	Dumeng	273	625	279	296	445	547	102	97	2319	2728	531	506
	Zhaozhou	308	469	241	238	290	478	70	144	1260	1804	615	407
	Fuyu	843	669	347	413	599	390	161	104	2217	2286	515	613
	Mingshui	561	605	375	448	584	432	144	122	2184	2222	462	625
Symmetric COO- stretching	Lanling	131	173	101	171	198	109	287	332	209	210	2648	2621
	Zhaodong	1174	1135	1119	1016	996	1782	469	589	239	272	3355	2481
	Dumeng	361	1635	713	701	1374	1527	141	211	236	220	2860	2604
	Zhaozhou	610	562	683	567	815	1107	291	254	208	229	2737	2316

	**Fuyu**	963	976	823	1059	1128	970	286	394	241	169	2801	2731
	**Mingshui**	96	113	64	140	135	53	44	33	231	269	2330	2433
**Si-O-Si & C-O stretching & O-H bending**	**Lanling**	3811	5368	4595	5184	5329	4940	3436	3380	3172	2922	5265	3094
	**Zhaodong**	3850	4271	4313	4216	3381	4873	3002	3913	3083	3561	2039	2530
	**Dumeng**	4344	5487	4881	4409	5717	5396	4889	5243	3217	2899	2123	3791
	**Zhaozhou**	4285	5146	4766	4536	5414	5542	2981	2915	3605	2835	3007	3002
	**Fuyu**	6280	5312	4991	5067	4901	4971	4301	2950	3356	2823	3722	2393
	**Mingshui**	4939	4924	4567	5662	5289	5822	4197	3902	3586	2996	4220	4884
**Carbonates**	**Lanling**	255	318	451	359	484	350	649	613	156	134	103	184
	**Zhaodong**	237	254	301	260	183	302	517	618	129	166	81	219
	**Dumeng**	418	218	353	282	332	379	974	1098	140	176	82	161
	**Zhaozhou**	286	378	410	325	391	447	480	451	224	131	81	83
	**Fuyu**	449	403	322	410	287	341	758	563	152	176	119	114
	**Mingshui**	294	349	460	481	350	586	662	554	156	97	61	92

Note: particulate fraction [PT], sand and aggregates [SA], soluble fraction [SB], acid insoluble fraction [AI], and silt and clay, i.e. acid-insoluble and easily oxidized fraction [AI+EO]

Table S3. The relative crystallinity of minerals in the bulk soil and soil fractions between the forest and the farmland

Minerals (%)	Regions	Bulk soil		AI+EO		AI		SA		PT		SB	
		Forest	Farmland	Forest	Farmland	Forest	Farmland	Forest	Farmland	Forest	Farmland	Forest	Farmland
Smectite-Vermiculite	Lanling	5.47	2.98	3.92	3.72	5.29	2.57	0.66	0.22	11.79	9.81	29.62	—
	Zhaodong	3.24	4.31	3.70	4.43	5.79	5.37	0.84	0.41	14.09	10.47	—	—
	Dumeng	2.04	1.16	2.74	3.76	10.94	9.74	0.33	—	13.04	9.51	—	—
	Zhaozhou	2.17	1.97	4.50	4.40	5.57	6.43	0.34	0.76	7.34	9.37	23.52	—
	Fuyu	6.31	4.48	5.65	8.13	14.92	8.59	0.74	2.44	13.23	11.22	—	—
	Mingshui	8.69	6.94	6.07	4.78	7.09	4.28	0.65	5.09	8.94	5.85	—	20.68
Quartz	Lanling	7.00	7.51	8.92	8.34	7.81	8.17	7.88	10.17	5.76	9.18	24.62	7.64
	Zhaodong	7.21	6.15	6.57	7.72	7.22	8.07	7.89	10.89	9.25	8.87	—	10.25
	Dumeng	5.04	8.78	8.56	9.23	7.44	5.43	20.50	10.72	13.17	8.20	36.09	—
	Zhaozhou	6.50	6.45	9.46	6.72	7.56	7.97	7.31	9.31	8.31	7.10	4.55	—
	Fuyu	9.05	8.38	7.69	7.25	6.74	5.93	7.84	10.63	13.58	9.29	—	—
	Mingshui	9.58	6.56	9.72	7.58	7.71	8.71	10.51	9.02	13.81	7.22	—	16.42
Cristobalite	Lanling	2.29	1.63	3.29	2.72	2.08	2.86	1.50	1.67	4.40	—	—	—
	Zhaodong	1.34	5.87	2.00	2.16	2.71	2.80	2.06	2.02	—	—	—	—
	Dumeng	2.23	0.79	2.66	2.66	4.67	2.01	1.10	1.07	3.86	4.22	—	6.18
	Zhaozhou	1.67	3.21	3.10	1.82	2.42	2.59	1.24	2.38	3.80	2.62	—	—
	Fuyu	2.84	1.87	1.87	2.10	2.78	2.45	1.45	0.74	—	8.18	—	—
	Mingshui	3.10	—	2.23	5.33	2.35	2.36	1.86	2.04	3.34	3.09	5.32	—
Quartz+Illinite	Lanling	42.71	40.32	44.20	42.11	36.03	33.13	38.28	43.48	40.23	40.63	—	—
	Zhaodong	34.64	38.09	39.97	39.25	36.49	41.30	33.73	49.02	33.79	33.15	—	—
	Dumeng	59.30	50.54	36.13	37.97	36.73	32.09	32.57	47.28	42.32	29.95	—	—
	Zhaozhou	41.99	35.41	43.39	41.67	40.77	41.16	45.63	43.53	37.14	41.54	—	—

	**Fuyu**	50.53	46.71	45.68	36.2	38.02	35.45	61.97	47.19	46.66	28.40	—	—
	**Mingshui**	37.41	53.29	43.67	36.49	39.98	43.8	39.03	44.49	37.29	23.95	17.52	—
**Feldspar**	**Lanling**	8.28	6.63	11.42	13.02	9.23	14.15	15.58	9.49	11.54	11.79	—	11.56
	**Zhaodong**	10.53	6.32	10.33	6.52	9.38	10.31	6.58	7.62	9.59	—	—	—
	**Dumeng**	13.81	7.70	11.47	7.45	12.98	10.52	6.97	6.54	9.32	14.74	—	—
	**Zhaozhou**	7.26	8.76	6.16	10.76	11.87	15.69	8.94	12.20	6.63	14.33	—	3.27
	**Fuyu**	4.84	4.07	0.85	6.62	7.07	9.13	10.95	4.58	12.11	9.69	—	14.50
	**Mingshui**	10.80	4.57	18.43	15.17	12.41	18.85	13.26	14.39	8.75	7.41	15.04	—
**Calcite**	**Lanling**	2.69	1.59	0.34	1.62	0.81	0.67	0.62	1.91	3.47	3.04	—	21.14
	**Zhaodong**	12.00	6.74	6.83	8.29	11.25	7.94	5.43	4.57	14.82	12.68	60.19	46.40
	**Dumeng**	1.36	2.95	4.70	6.55	9.74	9.38	1.94	5.28	7.61	3.34	39.35	63.55
	**Zhaozhou**	3.70	4.81	2.92	3.58	8.26	5.400	1.52	3.95	7.81	4.64	36.59	47.24
	**Fuyu**	4.96	6.11	6.33	9.14	9.95	8.82	2.18	1.06	5.36	8.57	31.95	—
	**Mingshui**	4.56	2.12	5.48	2.64	2.89	2.11	3.46	3.23	3.93	2.73	—	4.71
**Huntite**	**Lanling**	0.83	1.11	0.66	0.61	—	0.48	0.20	0.74	—	—	20.38	16.9
	**Zhaodong**	—	0.48	0.56	0.43	0.71	0.76	0.57	0.50	—	—	28.42	3.83
	**Dumeng**	0.22	0.27	0.81	—	—	0.95	0.38	0.22	—	—	—	15.26
	**Zhaozhou**	0.25	0.73	0.65	0.50	0.38	—	0.27	0.19	1.73	—	35.33	20.67
	**Fuyu**	0.36	0.70	—	—	—	—	—	—	—	—	28.71	48.97
	**Mingshui**	0.84	—	0.64	—	0.81	—	0.44	—	—	1.98	18.84	21.13

Note: particulate fraction [PT], sand and aggregates [SA], soluble fraction [SB], acid insoluble fraction [AI], and silt and clay, i.e. acid-insoluble and easily oxidized fraction [AI+EO]
